# Lymphocyte subset alterations with disease severity, imaging manifestation, and delayed hospitalization in COVID-19 patients

**DOI:** 10.1186/s12879-021-06354-7

**Published:** 2021-07-01

**Authors:** Daxian Wu, Xiaoping Wu, Jiansheng Huang, Qunfang Rao, Qi Zhang, Wenfeng Zhang

**Affiliations:** 1 Department of Infectious Diseases, the First Affiliated Hospital, Nanchang University, No.17 Yongwai Street, Donghu District, Nanchang, 330006 Jiangxi Province China; 2 Department of Infectious Diseases, Nanfang Hospital, Southern Medical University, Guangzhou, 510515 China

**Keywords:** COVID-19, Delayed hospitalization, Computed tomography, Lymphocyte subsets, Flow cytometry

## Abstract

**Background:**

COVID-19 continuously threated public health heavily. Present study aimed to investigate the lymphocyte subset alterations with disease severity, imaging manifestation, and delayed hospitalization in COVID-19 patients.

**Methods:**

Lymphocyte subsets was classified using flow cytometry with peripheral blood collected from 106 patients.

**Results:**

Multivariate logistic regression showed that chest tightness, lymphocyte count, and γ-glutamyl transpeptidase were the independent predictors for severe COVID-19. The T cell, CD4^+^ T cell and B cell counts in severe patients were significantly lower than that in mild patients (*p* = 0.004, 0.003 and 0.046, respectively). Only the T cell count was gradually decreased with the increase of infiltrated quadrants of lesions in computed tomography (CT) (*p* = 0.043). The T cell, CD4^+^ T cell, and CD8^+^ T cell counts were gradually decreased with the increase of infiltrated area of the maximum lesion in CT (*p* = 0.002, 0.003, 0.028; respectively). For severe patients, the counts of T cell, CD4^+^ T cell, CD8^+^ T cell gradually decreased with the increased delayed hospitalization (*p* = 0.001, 0.03, and <  0.001, respectively). The proportions of T cell, CD8^+^ T cell gradually decreased with the increased delayed hospitalization (both *p* <  0.001), but the proportions of NK cell, B cell gradually increased with the increased delayed hospitalization (*p* = 0.007, and 0.002, respectively). For mild patients, only the NK cell count was gradually decreased with the increased delayed hospitalization (*p* = 0.012).

**Conclusion:**

T lymphocyte and its subset negatively correlated with disease severity, CT manifestation and delayed hospitalization. The counts of lymphocyte subset were changed more profound than their proportions.

## Introduction

Coronavirus disease 2019 (COVID-19) is a newly emerged viral infection caused by the severe acute respiratory syndrome coronavirus 2 (SARS-CoV-2) [1]. COVID-19 is highly contagious and has become pandemic quickly. Innate and adaptive immune responses are activated in COVID-19 patients, perhaps uncontrolled innate and adaptive immune responses may lead to locally and systemically tissue damage. Recently, the alterations of lymphocyte subsets in COVID-19 patients had attracted the attention of researchers for exhausted lymphocytes were a feature of severe COVID-19 [2–4]. An overall decline of lymphocyte subsets including CD4^+^ T cells, CD8^+^ T cells, B cells, and NK cells has been reported in severe and deceased COVID-19 patients [5, 6]. However, vary patterns of lymphocyte subsets abnormality in severe COVID-19 patients also have been demonstrated by other studies [7–10]. Reports involving the change of CD4^+^ to CD8^+^ T cells ratio were also inconsistent [9, 11, 12]. Thus, the reported patterns of lymphocyte subsets in patients with COVID-19 were diverse and controversial, and necessitated to clear more.

Lung computed tomography (CT) plays an important role in the early diagnosis and evaluating the disease severity of COVID-19 for different imaging manifestations are demonstrated at different stages of the disease [13]. At the early stages of the COVID-19, unilateral or bilateral ground-glass opacity (GGO) is most common in the posterior aspects and periphery of the lungs. With the progression of the disease, the scope and number of GGO are gradually expanding and fusion. At the later stages of the COVID-19, crazy paving appearance and pulmonary consolidation begin to appear and are gradually extensive. As far, the correlation of lymphocyte subsets with the lesion manifestation in lung CT was not be well documented and necessitated to clarify.

Here, we first investigated alterations of lymphocyte subsets in severe COVID-19 patients. Then, we observed the correlations of lymphocyte subsets with the number, quadrant, and area of lesions in lung CT. Finally, we investigated the impacts of the lymphocyte subsets in patients with delayed hospitalization.

## Method

### Patients

One hundred and six COVID-19 patients who were confirmed by positive RNA of SARS-CoV-2 using throat swab specimens were prospectively recruited from June 23, 2020 to February 29, 2020 at the First Affiliated Hospital, Nanchang University. Patients were stratified at their admission, 33 patients with severe COVID-19 was diagnosed according the guideline of the American Thoracic Society and Infectious Diseases Society of America [14]. 73 cases not meeting the criteria were classified as mild COVID-19. All procedures followed were in accordance with the Ethics Committees of the First Affiliated Hospital, Nanchang University, and with the Helsinki Declaration of 1975, as revised in 2000. All patients enrolled in study were over 16 years old, and written informed consent was obtained from themselves or their legal representatives.

### Data acquisition

Data on the demography, epidemiology, symptoms and signs, laboratory tests, as well as radiography findings were extracted from electronic medical records using a predesigned datasheet. All laboratory tests were conducted in the Central Clinical Laboratory of the First Affiliated Hospital, Nanchang University and were adopted if they were performed with fasting blood samples at patients’ admission.

### Flow cytometry

Anticoagulated peripheral blood samples with EDTA were collected from COVID-19 patients at their admission and tested within 6 h. Lymphocyte subsets was performed by Cytomics FC 500 flow cytometer and analysed by CXP Analysis software (Beckman Coulter, Brea, California). Anti-CD3 was conjugated by PE-texas red (ECD), anti-CD4, anti-CD8, anti-CD19 were conjugated by fluorescein isothiocyanate (FITC), PE-Cy5 (PC5), and R-phycoerythrin (PE) respectively. The count of NK cell marked by CD3^−^CD16^+^CD56^+^ was auto calculated CXP Analysis software. All tests were performed according to the manufacturer’s instructions.

### Statistics

Statistical analysis was performed with SPSS 25.0 (SPSS, Inc., Chicago, USA) and MedCalc (MedCalc Software Ltd., Ostend, Belgium). Continuous data were expressed as the mean ± standard deviations or medians with quartile (P25-P75) and categorical data were expressed as numbers (%). The Student’s t-test was used for continuous data distributed normally, and the Mann–Whitney U test was used for continuous data distributed abnormally. The χ2 or Fisher’s tests were used for categorical data. Rank correlation was analysed using the Spearman method. A *p* values of less than 0.05 was considered statistically significant. Independent risk factors were identified using multivariate logistic regression according to the forward Wald method, with entry and removal probabilities of 0.05 and 0.10, respectively.

## Results

### Baseline clinical characteristics of patients with COVID-19

The age of patients with COVID-19 was 46.17 ± 14.39, and 60.4% patients were male. The mean of the time from onset to hospitalization (TOH) was 5 days. Among the 106 COVID-19 patients, 83 (78.3%) patients had a clear exposure history, and 33 (31.1%) patients had one or more comorbidities. The most frequent comorbidity was bacterial infection (11.3%), followed by diabetes (9.4%) and hypertension (7.5%). As expected, fever (91.5%), dry cough (43.4%), and chest tightness (32.1%) were the top three frequent symptoms. Chills (20.8%), fatigue (19.8%), and sore throat (18.9%) were also common, but rhinorrhea or rhinobyon (5.7%), diarrhea (6.6%), and myalgia (7.5%) were relatively rare in COVID-19 patients (Table 1).

**Table 1:** Characteristics at admission of the patients with COVID-19

Variable	Total (***n*** = 106)	Mild (***n*** = 73)	Severe (***n*** = 33)	Univariate logistic regression		Multivariate logistic regression	
				HR (95% CI)	P	HR (95% CI)	P
**Epidemiological and clinical characteristics**							
**Age (years)**	46.17 ± 14.39	44.95 ± 13.59	48.88 ± 15.88	1.020 (0.990–1.050)	0.194		
**Gender (female/male)**	42/64	31/42	11/22	1.476 (0.625–3.488)	0.375		
**Time from onset to hospitalization (d)**	5 (2–8)	4 (2–7)	7 (4–10)	1.156 (1.034–1.293)	0.011		
**Exposure history(Y/N)**	83 (78.3%)	55 (75.3%)	28 (84.8%)	1.833 (0.616–5.453)	0.276		
**Any comorbidities**	33 (31.1%)	18 (24.7%)	15 (45.5%)	2.546 (1.069–6.063)	0.035		
**Hypertension**	8 (7.5%)	4 (5.5%)	4 (12.1%)	2.379 (0.557–10.166)	0.242		
**Diabetes**	10 (9.4%)	6 (8.2%)	4 (12.1%)	1.540 (0.404–5.871)	0.527		
**Hepatitis B**	7 (6.6%)	6 (8.2%)	1 (30%)	0.349 (0.040–3.021)	0.339		
**Bacterial infection**	12 (11.3%)	7 (9.6%)	5 (15.2%)	1.684 (0.492–5.759)	0.406		
**Signs and symptoms**							
**Fever**	97 (91.5%)	66 (90.4%)	31 (93.9%)	1.644 (0.323–8.377)	0.550		
**Dry cough**	46 (43.4%)	30 (41.1%)	16 (48.5%)	1.349 (0.590–3.084)	0.478		
**Sputum production**	12 (11.3%)	5 (6.8%)	7 (21.2%)	3.662 (1.067–12.570)	0.039		
**Chills**	22 (20.8%)	17 (23.3%)	5 (15.2%)	0.588 (0.197–1.759)	0.342		
**Myalgia**	8 (7.5%)	6 (8.2%)	2 (6.1%)	0.720 (0.138–3.774)	0.698		
**Chest tightness**	34 (32.1%)	15 (20.5%)	19 (57.6%)	5.248 (2.147–12.827)	< 0.001	3.256 (1.134–9.345)	0.028
**Polypnea**	14 (13.2%)	4 (5.5%)	10 (30.3%)	7.500 (2.145–26.227)	0.002		
**Fatigue**	21 (19.8%)	12 (16.4%)	9 (27.3%)	1.906 (0.712–5.104)	0.199		
**Headache/ dizziness**	13 (12.3%)	12 (16.4%)	1 (3.0%)	0.159 (0.020–1.277)	0.084		
**Sore throat**	20 (18.9%)	14 (19.2%)	6 (18.2%)	0.937 (0.325–2.701)	0.903		
**Rhinorrhea/ Rhinobyon**	6 (5.7%)	5 (6.8%)	1 (3.0%)	0.425 (0.048–3.789)	0.443		
**Diarrhea**	7 (6.6%)	4 (5.5%)	3 (9.1%)	1.725 (0.364–8.185)	0.493		
**Laboratory parameters**							
**CRP (mg/L)**	12.63 (3.53–40.49)	9.00 (2.46–24.93)	22.99 (6.36–77.01)	1.011 (1. 002–1.021)	0.018		
**WBC (×10**^**9**^**/L)**	5.14 (3.63–6.79)	4.84 (3.60–6.63)	5.68 (3.68–7.00)	1.076 (0.926–1.249)	0.339		
**Lymphocyte count (× 10**^**9**^**/L)**	0.99 (0.65–1.40)	1.13 (0.86–1.58)	0.68 (0.40–0.99)	0.065 (0.018–0.244)	< 0.001	0.097 (0.024–0.396)	0.010
**Neutrophils count (× 10**^**9**^**/L)**	3.47 (2.30–5.17)	3.19 (2.07–4.89)	3.95 (2.66–5.95)	1.163 (0.996–1.357)	0.055		
**RBC (× 10**^**12**^**/L)**	4.61 ± 0.55	4.66 ± 0.54	4.50 ± 0.57	0.571 (0.262–1.241)	0.157		
**Hemoglobin (g/L)**	143.59 ± 16.91	144.29 ± 17.37	142.06 ± 16.02	0.992 (0. 969–1.016)	0.530		
**Platelets (× 10**^**9**^**/L)**	176.16 ± 62.11	181.53 ± 61.09	164.27 ± 63.65	0.995 (0. 987–1.003)	0.189		
**Albumin (g/L)**	43.03 ± 6.07	44.32 ± 5.71	40.16 ± 5.94	0.857 (0.781–0.939)	0.001		
**ALT (U/L)**	18.00 (12.00–34.00)	16.00 (12.00–30.50)	23.00 (16.00–43.00)	1.010 (0.996–1.025)	0.167		
**AST (U/L)**	24.00 (19.00–33.00)	22.00 (18.00–28.50)	28.00 (22.00–36.50)	1.010 (0.994–1.027)	0.228		
**Total bilirubin (μmol/L)**	9.05 (5.55–12.38)	7.70 (5.30–11.40)	9.60 (7.20–15.40)	1.054 (0.982–1.130)	0.143		
**Direct bilirubin (**μmol**/L)**	2.60 (2.00–4.03)	2.50 (1.95–3.60)	3.60 (2.40–5.65)	1.198 (1.024–1.401)	0.024		
**GGT (U/L)**	24.00 (14.00–47.50)	20.00 (12.00–34.00)	41.00 (22.00–71.50)	1.011 (1.001–1.022)	0.034	1.011 (1.002–1.021)	0.022
**Lactate dehydrogenase**	231.50 (190.75–320.25)	211.00 (183.50–269.50)	291.00 (239.50–394.50)	1.008 (1.004–1.013)	0.001		
**Creatinine (mmol/L)**	65.20 (52.00–79.90)	65.70 (51.85–79.50)	63.60 (52.70–81.58)	1.004 (0.987–1.020)	0.668		
**Urea nitrogen (mmol/L)**	4.20 (3.40–5.35)	4.10 (3.30–5.30)	4.55 (3.53–5.50)	1.068 (0.879–1.298)	0.507		
**Creatine kinase (U/L)**	85.00 (59.25–125.00)	85.00 (60.00–124.50)	97.00 (51.00–134.00)	1.003 (0.998–1.007)	0.270		
**CK-MB (U/L)**	13.00 (10.00–16.00)	13.00 (10.00–15.00)	13.00 (10.00–19.00)	1.017 (0.985–1.050)	0.297		
**Prothrombin time (s)**	12.30 (11.90–12.85)	12.25 (11.83–12.70)	12.40 (12.10–13.15)	1.247 (0.769–2.022)	0.371		
**Thrombin time (s)**	15.50 (14.95–16.40)	15.60 (15.10–16.65)	15.30 (14.65–16.15)	0.975 (0.707–1.344)	0.877		
**APTT (s)**	29.30 (27.25–32.95)	29.20 (27.13–32.38)	30.30 (27.80–33.75)	1.004 (0.951–1.060)	0.879		
**D-dimer (mg/L)**	0.29 (0.19–0.57)	0.25 (0.16–0.50)	0.49 (0.27–1.03)	3.883 (1.485–10.151)	0.006		

*Abbreviations: CRP* C-reactive protein, *WBC* white blood cell count, *RBC* red blood cell count, *ALT* alanine aminotransferase, *AST* aspartate aminotransferase, GGT γ-glutamyl transpeptidase, *APTT* activated partial thromboplastin time

### Independent indictors for severe COVID-19

As shown in Table 1, univariate logistic regression indicated that the TOH of severe patients was significantly longer than of mild patients [7 (4–10) vs. 4 (2–7) days; *p* = 0.011]. The frequency of comorbidities in severe patients was higher than mild patients (45.5% vs. 24.7%; *p* = 0.035). The frequency of sputum production, chest tightness, or polypnea was higher in severe patients than mild patients (all *p* <  0.05). The level of C-reactive protein, as well as levels of direct bilirubin, γ-glutamyl transpeptidase, lactate dehydrogenase, and D-dimer were significantly higher in severe patients than mild patients (all p <  0.05). However, the levels of lymphocyte count and albumin were significantly lower in severe cases compared to mild cases. Multivariate logistic regression showed that chest tightness, lymphocyte count, and γ-glutamyl transpeptidase were the independent indictors to predict severe COVID-19.

### Lymphocyte subsets in severe COVID-19 patients

**Fig. 1:**
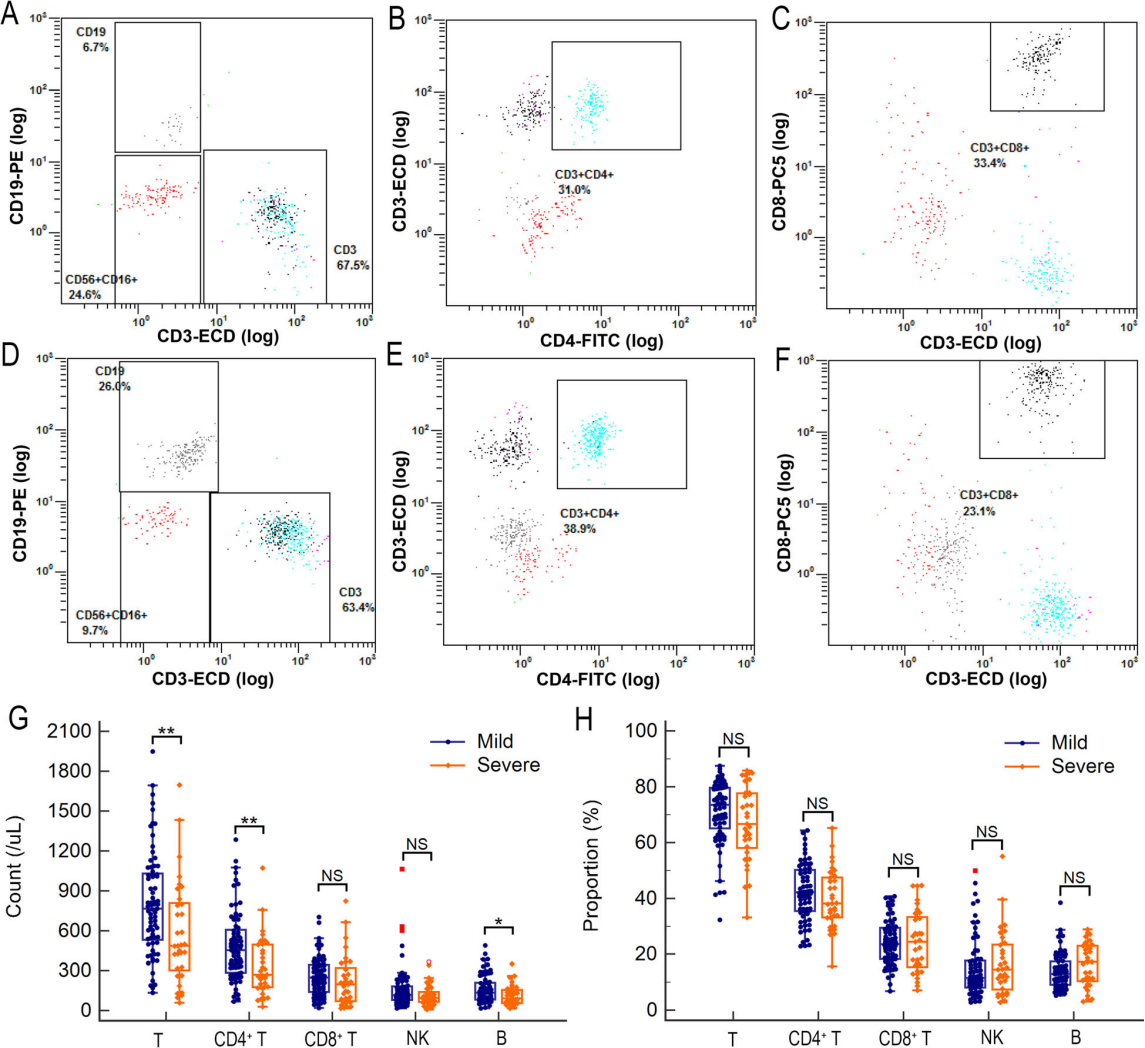
The lymphocyte subset alterations in severe COVID-19 patients. Representative flow cytometry plots for T cell, B cell, and NK cell (**A**), CD4^+^ T cell (**B**), and CD8^+^ T cell (**C**) for severe COVID-19 patients. Representative flow cytometry plots for T cell, B cell, and NK cell (**E**), CD4^+^ T cell (**F**), and CD8^+^ T cell (**G**) for mild COVID-19 patients. The comparison of absolute counts (**G**) and proportions (**H**) of lymphocyte subset between severe and mild COVID-19 patients. The differences were compared using the Mann-Whitney *U* test. *P < 0.05 and **P < 0.01; NS, not significant

Giving the lymphocyte count was an important indicator to predict severe COVID-19, we further investigated the alteration of lymphocyte subsets in patients with severe COVID-19. As shown in Fig. 1A, the T cell count in severe patients was significantly lower than that in mild patients [487.00 (291.50, 819.50) vs. 766.00 (525.50, 1036.50) /μL; *p* = 0.004]. The CD4^+^ T cell and B cell counts in severe patients were also significantly lower than that in mild patients [272.00 (177.00, 497.50) vs. 455.00 (283.50, 612.50) and 92.00 (57.50, 160.00) vs. 136.00 (82.50, 213.00) /μL; *p* = 0.003 and 0.046, respectively]. There is no significant difference for CD8^+^ T cell or NK cell count between severe and mild patients. The difference of CD4^+^ to CD8^+^ ratio between severe and mild patients was not significant. No significant difference for proportion of lymphocyte subset was observed between severe and mild patients (Fig. 1B).

### The basic lymphocyte status of patients with comorbidities

**Fig. 2:**
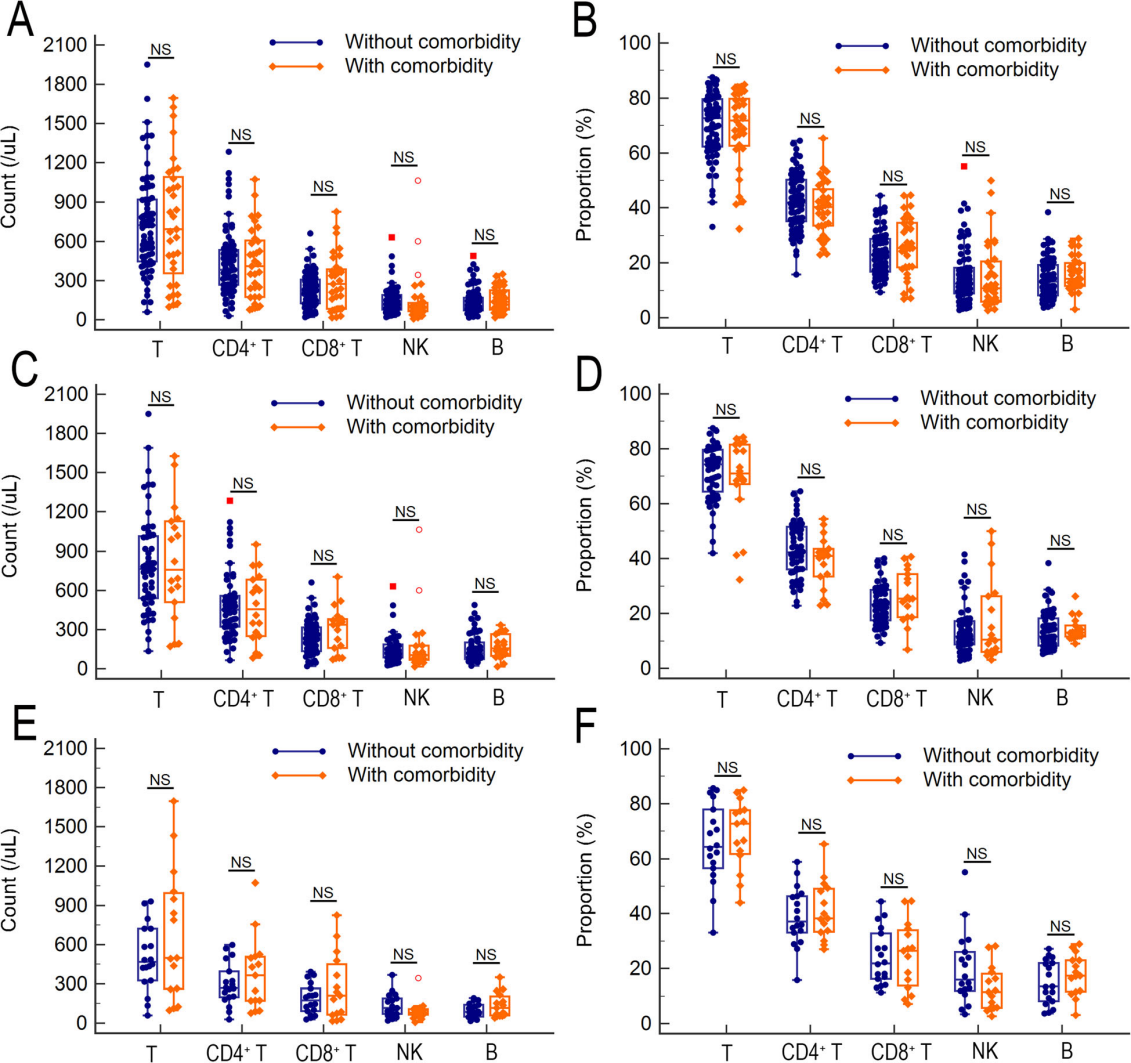
Association of lymphocyte subset alterations with comorbidity in COVID-19 patients. The count (**A**) and proportion (**B**) of lymphocyte subset differences between COVID-19 patients with and without comorbidity. The count (**C**) and proportion (**D**) of lymphocyte subset differences between mild COVID-19 patients with and without comorbidity. The count (**E**) and proportion (**F**) of lymphocyte subset differences between severe COVID-19 patients with and without comorbidity. The differences were compared using the Mann-Whitney *U* test. NS, not significant

As described above, patients with comorbidities were prone to severe COVID-19, and the decreased T cell, CD4^+^ T cell and B cell counts were closed related to severe COVID-19. Thus, we further investigated that whether the patients with comorbidities were prone to severe COVID-19 related to their basic lymphocyte status? As shown in Fig. 2, there are no significant differences between COVID-19 patients with comorbidity and who without comorbidity, no matter for the counts or the proportions of all lymphocyte subsets (Fig. 2A-B). The same result was obtained when the COVID-19 patients were stratified with disease severity (Fig. 2C-F). Those results suggested that the reason why patients with comorbidities were prone to severe COVID-19 were independent of their basic lymphocyte status.

### Lymphocyte subsets alterations with CT manifestation

**Fig. 3:**
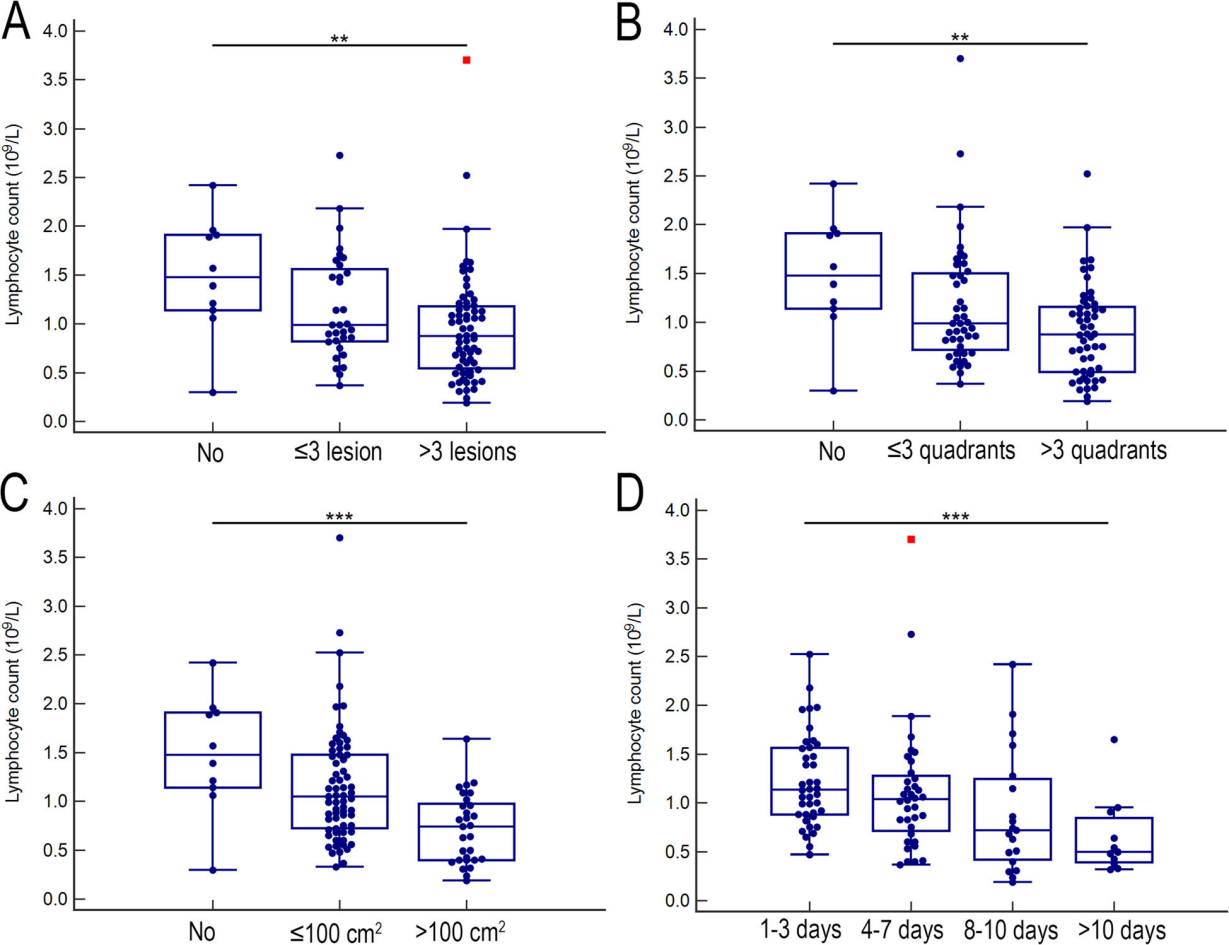
The total lymphocyte cell count with aggravated CT manifestation and increased delayed hospitalization. The lymphocyte count was gradually decreased with the increased number (**A**), quadrant (**B**), or area (**C**) of lesions. The lymphocyte count was gradually decreased with the increased delayed hospitalization (**D**). The trend tests were analyzed with rank correlation using the Spearman method. **P < 0.01, and ***P < 0.001

**Fig. 4:**
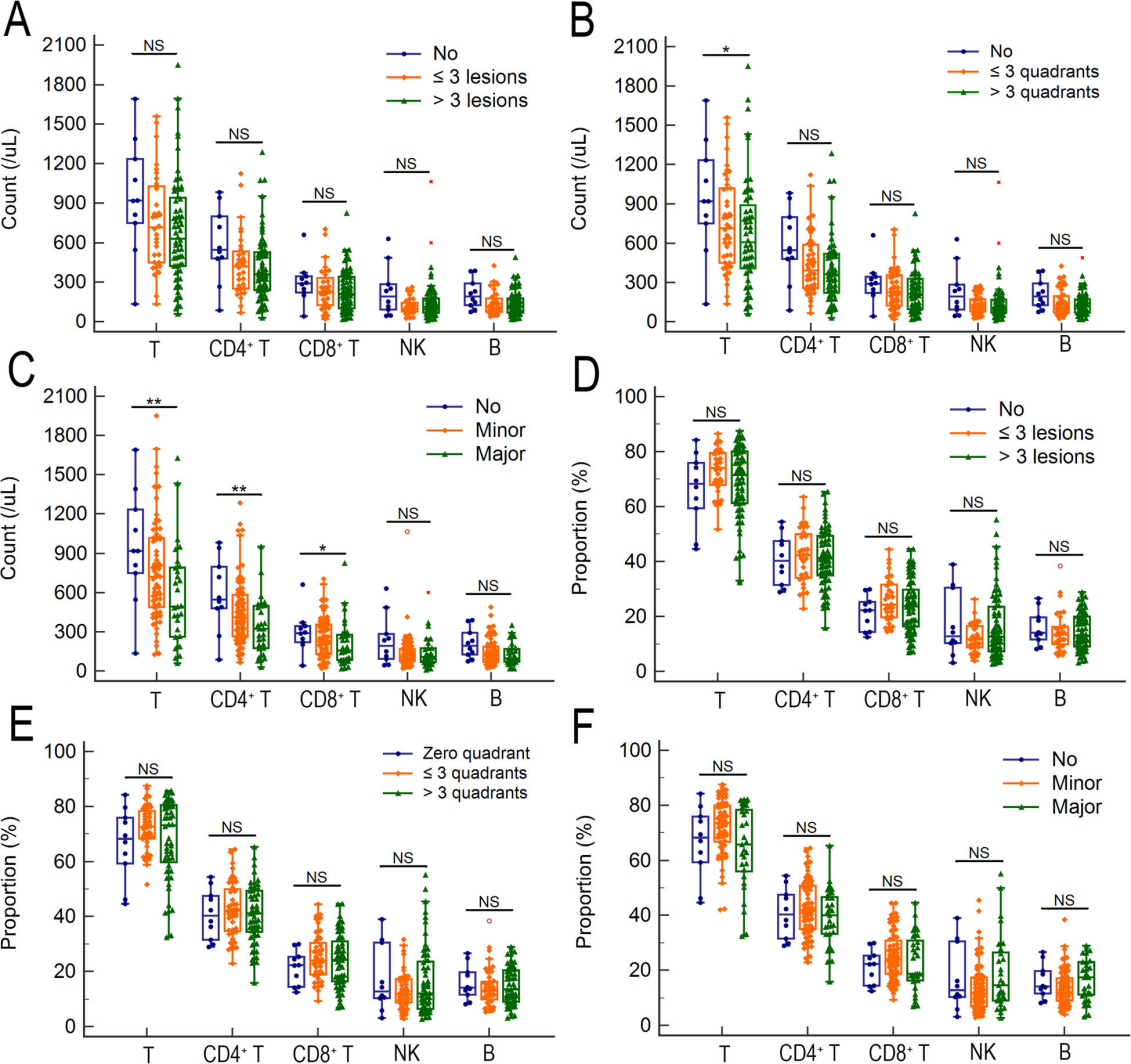
The lymphocyte subset alterations with aggravated CT manifestation. The count alterations of lymphocyte subset with the increased number (**A**), quadrant (B), or area (**C**) of lesions. The proportion alterations of lymphocyte subset with the increased number (**D**), quadrant (**E**), or area (**F**) of lesions. The trend tests were analyzed with rank correlation using the Spearman method. *P < 0.05, **P < 0.01, and ***P < 0.001; NS, not significant

In order to assess the manifestation of lesions in lung CT, present study simply scored the number, quadrant, and area of lesions. For the number of lesions, patients were classified to 3 subgroups named patients with no lesion, ≤3 lesions, and > 3 lesions. For the quadrant of lesions, patients were classified to 3 subgroups, that is, patients with no quadrant, ≤3 quadrants, and > 3 quadrants. For the area of the maximum lesion, patients were classified no infiltration when there is no lesion in CT. patients with minor and major infiltration were classified when the area of the maximum lesion were ≤ 100 cm^2^ and > 100 cm^2^ respectively. As shown in Fig. 3A-C, the lymphocyte counts were gradually decreased with the increased number, quadrant, or area of lesions (*p* = 0.002, 0.002, and < 0.001 respectively). No significant trend of absolute count of any lymphocyte subset was observed with the increase of lesion number (Fig. 4A). Only the T cell count was gradually decreased with the increase of infiltrated quadrants (*p* = 0.043) (Fig. 4B). The T cell, CD4^+^ T cell, and CD8^+^ T cell counts were gradually decreased with the increase of infiltrated area (*p* = 0.002, 0.003, 0.028, respectively) (Fig. 4C). However, the trend of CD4^+^ to CD8^+^ ratio was not significant no matter with the increase of the number, quadrant, or the area of lesions. For proportion, there is also no significant trend of any lymphocyte subset with aggravated CT manifestation (Fig. 4D-F).

### Lymphocyte subsets alterations with TOH

**Fig. 5:**
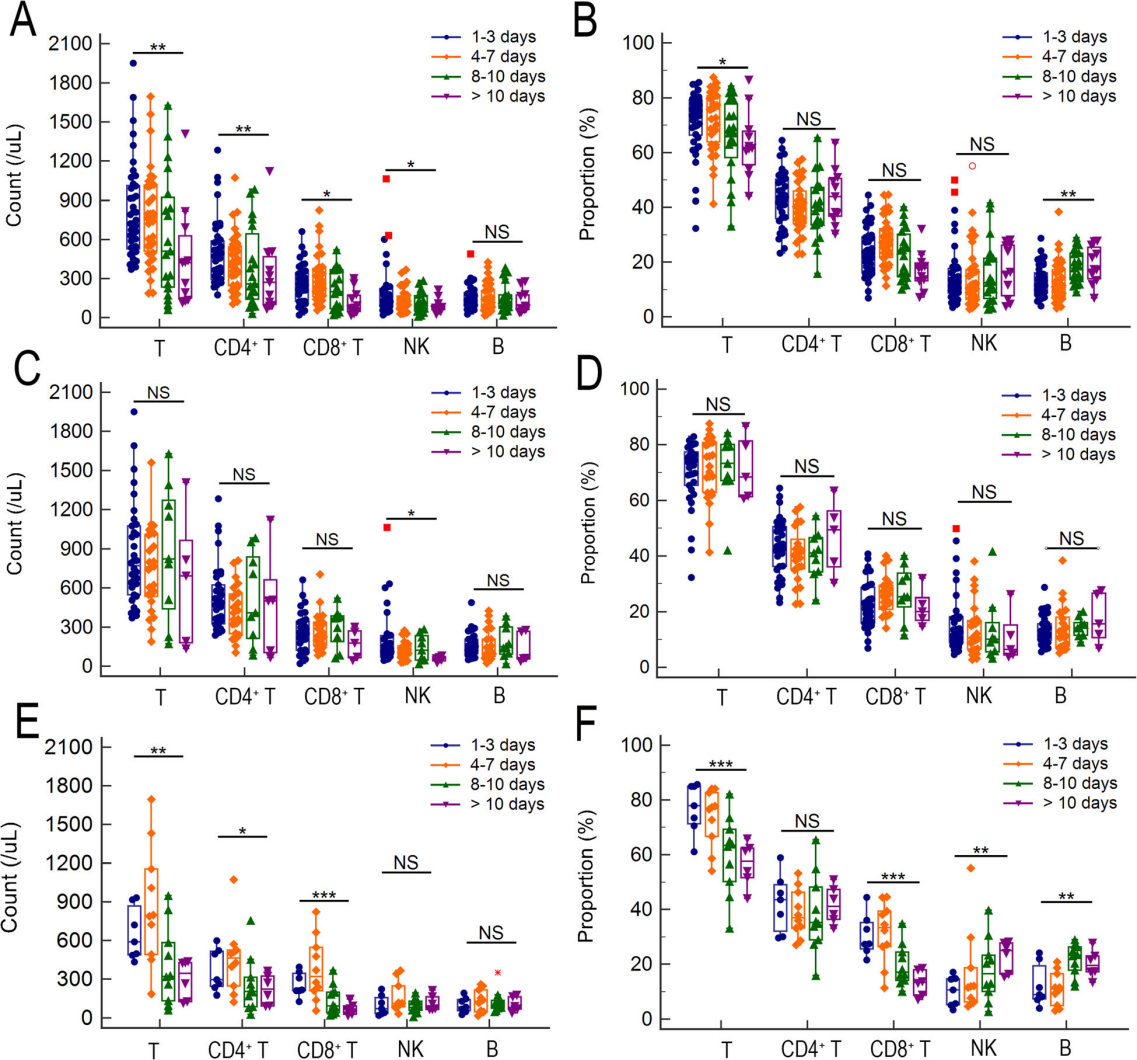
The lymphocyte subset alterations with increased delayed hospitalization. The alterations of absolute counts (**A**) and proportions (**B**) of lymphocyte subset with increased delayed hospitalization. The alterations of absolute counts (**C**) and proportions (**D**) of lymphocyte subset in mild patients with increased delayed hospitalization. The alterations of absolute counts (**E**) and proportions (**F**) of lymphocyte subset in severe patients with increased delayed hospitalization. The trend tests were analyzed with rank correlation using the Spearman method. *P < 0.05, **P < 0.01, and ***P < 0.001; NS, not significant

The lymphocyte counts were gradually decreased with the increased TOH (*p* < 0.001, Fig. 3D). Lymphocyte subsets analysis showed that the T cell count, as well as CD4^+^ T cell, and CD8^+^ T cell counts were gradually decreased with the increased TOH (*p* = 0.003, 0.002, and 0.013, respectively). The NK cell count was also gradually decreased with the increased TOH (*p* = 0.012) (Fig. 5A). There is no significant trend of CD4^+^ to CD8^+^ ratio with the delayed hospitalization. The proportion of T cell was gradually decreased with the increased TOH (*p* = 0.031), but the proportion of B cell was gradually increased with the increased TOH (*p* = 0.003) (Fig. 5B).

When stratified the patients by disease severity, for mild COVID-19 patients, only the NK cell count was also gradually decreased with the increased TOH (p = 0.012) (Fig. 5C). As shown in Fig. 5E-F, for severe COVID-19 patients, both the absolute count and proportion of T cell were gradually decreased with the increased TOH (*p* = 0.001 and < 0.001, respectively), as well as the CD8^+^ cell (both *p* < 0.001). The count of CD4^+^ cell was gradually decreased with the increased TOH (*p* = 0.03) but the proportion of CD4^+^ cell did not change with the increased TOH (*p* = 0.966). Conversely, the proportion of NK cell and B cell were gradually increased with the increased TOH (*p* = 0.007 and 0.002, respectively), but the counts of NK cell and B cell did not change with the increased TOH (*p* = 0.861 and 0.930, respectively).

### Organ injury with TOH

**Fig. 6:**
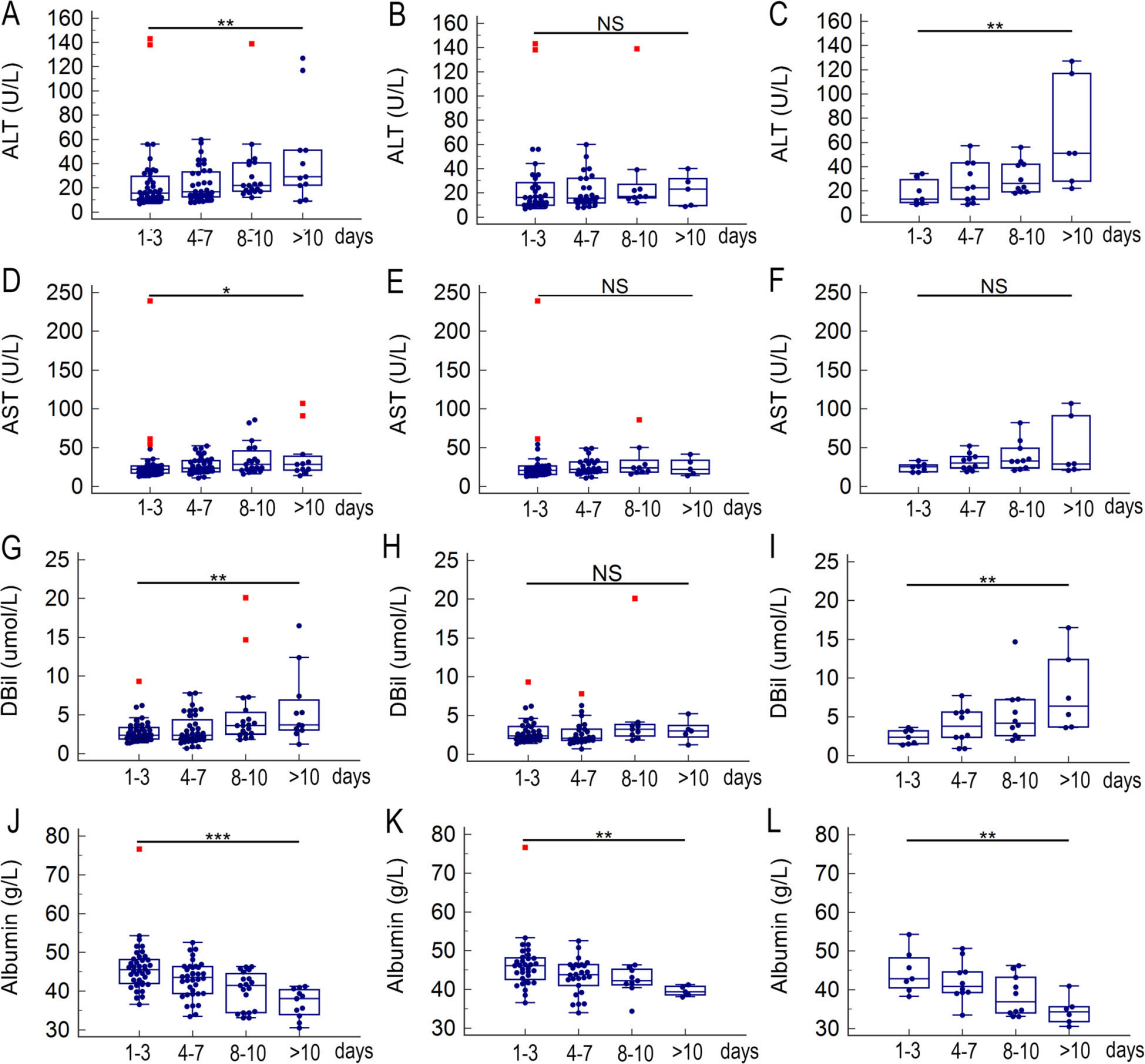
The alterations of parameters related to liver function with increased delayed hospitalization. The alteration of ALT level with increased delayed hospitalization in total (**A**), mild (**B**), and severe (**C**) COVID-19 patients; The alteration of AST level with increased delayed hospitalization in total (**D**), mild (**E**), and severe (**F**) COVID-19 patients; The alteration of DBil level with increased delayed hospitalization in total (**G**), mild (**H**), and severe (**I**) COVID-19 patients; The alteration of albumin level with increased delayed hospitalization in total (**J**), mild (**K**), and severe (**L**) COVID-19 patients. The trend tests were analyzed with rank correlation using the Spearman method. *P < 0.05, **P < 0.01, and ***P < 0.001; NS, not significant. Abbreviations: ALT, alanine aminotransferase; AST, aspartate aminotransferase; DBil, direct bilirubin

As shown in Fig. 6, for all enrolled COVID-19 patients, the levels of ALT, AST, and DBil were gradually increased with increased TOH (*p* = 0.004, 0.014 and 0.002, respectively), but level of albumin was gradually decreased with increased TOH (*p* < 0.001). Similar results were observed in severe patients but the AST level did not change with increased TOH (*p* = 0.098). For mild patients, only level of albumin was gradually decreased with increased TOH (*p* = 0.001). The level of TBil did not change with increased TOH, no matter for all enrolled, mild, or severe patients. Other indicators related to injures of kidney (creatinine, BUN), coagulation (PT, APTT), cardiac muscle (CK-MB), and skeletal muscle (CK) also did not change with increased TOH, no matter for all enrolled, mild, or severe patients (data not shown).

## Discussion

COVID-19 continuously threated public health heavily, which required more bench and clinic studies to profile this disease more profoundly. The common symptoms in COVID-19 patients, in accordance with previous reports [2, 15, 16], were fever, and followed by dry cough, and chest tightness. Similar with studies investigated in other area [17, 18], the age of patients in this study was dramatically younger than those in Wuhan. Notably, the frequencies of hypertension and diabetes for COVID-19 patients in this study were also much lower than patients in Wuhan [2, 15, 19]. The underlying reason may interpret by the lower age. Present study showed that there are 11.3% patients accompanying with bacterial infection, which suggested more attention should be paid to the evaluation of bacterial infection on patients’ admission. Additionally, present study found the risk of severe COVID-19 in patients with comorbidities is much higher (HR = 2.546) than those who without comorbidities. More importantly, present study found that the reason why patients with comorbidities were prone to severe COVID-19 were independent of their basic lymphocyte status (Fig. 2).

Complex immune dysregulation has been found in COVID-19 patients [20]. Currently, the change patterns of lymphocyte subsets were not conclusive. It is reported [5, 6] that a whole decline of lymphocyte subsets including CD4^+^ and CD8^+^ T cells, B cells, and NK cells were presented in severe and deceased COVID-19 patients. Liu et al. [10] suggested CD8^+^ T cell count was significant decreased in severe COVID-19 patients than mild patients at the time point of disease onset and 7–9 days later, but their difference in CD4^+^ T cell count was not significant at any time point. studies [7–9] also indicated decreased CD4^+^ and CD8^+^ T cells were correlated with disease severity of COVID-19, but there is no difference for the level of B or NK cell between severe and mild COVID-19 patients. Additionally, reports involving the change of CD4^+^ to CD8^+^ T cells ratio were also inconsistent [9, 11, 12]. In this study, we found the significant percentage change of lymphocyte subsets is rare no matter in patients with severer clinical type or more extensive CT manifestation. These findings were agreement with previous studies [4, 7]. It is noteworthy that the T cell and CD4^+^ T cell but not CD8^+^ T cell were significantly decreased in severe COVID-19 patents, which suggested that CD4^+^ T cell but not CD8^+^ T cell play more important role in immunity response to SARS-CoV-2 infection. Studies using SARS-CoV or MERS-CoV infected mouse demonstrated that depletion of CD4^+^ T cells but not CD8^+^ T cells would lead to delayed clearance of virus and enhanced immune-mediated pneumonitis [21, 22]. Similarly, high-level CD4^+^ but not the CD8^+^ T cell response was also observed in SARS patients [23]. What is more, the significantly decreased B cell in severe COVID-19 patents indicated that humoral immunity has been attenuated in antiviral response of SARS-CoV-2. It has reported [24–26] that T-helper type 1 (Th1), T-helper type 2 (Th2), and regulatory T cells were varying degrees of activated in peripheral blood from critical COVID-19 patients after stimulation with specific antigen of SARS-CoV-2. It can be speculated all the CD4^+^ T cell subgroups were exhaust in blood of critical COVID-19 patients for the severely damaged lymphoid organs and/or exudation of circulating lymphocytes into lung [9], although the alteration of CD4^+^ T cell subsets warrants further investigation.

With regard to lymphocyte subset changes with CT manifestation, present study found that the total lymphocyte counts were gradually decreased with the increased number, infiltrated quadrants of lesions, and the area of the maximum lesion. T cell counts were gradually decreased with the increase of infiltrated quadrants of lesions and the area of the maximum lesion rather than the increased lesion number. Gradually decreased CD4^+^ and CD8^+^ T cell counts were only observed with increased area of the maximum lesion. Those results revealed that the area of the maximum lesion was closer correlated with the count of lymphocyte subsets and was more appropriate to estimate the severity of COVID-19.

The alteration of lymphocyte subsets with the delayed hospitalization has not been reported before, present study firstly observed their correlation and found that the total lymphocyte, T cell, CD4^+^ and CD8^+^ T cell counts were gradually decreased with the the increased TOH for all enrolled COVID-19 patients. The same results were observed in severe patients but not observed in mild patients (Fig. 5). Those findings indicated that the lymphatic organs will continue to be damaged for severe patients if there is no intervention. Liver was a predominantly vulnerable extrapulmonary organ in patients with COVID-19, hepatic dysfunction was seen in 14–53% of cases and particularly in those with severe condition [27]. Similar with the trends of lymphocyte subsets, present study also found the level of ALT was gradually elevated with the TOH (Fig. 6), suggested that delayed hospitalization may cause more liver injure. Therefore, early hospitalization could avoid disease aggravation and the unnecessary use of scarce medical resources.

There were several limitations in this study. First, the alteration of CD4^+^ T cell subsets was not investigated, although CD4^+^ T cell was demonstrated to be mainstay of immunity response to severe SARS-CoV-2 infection. Second, only 3 cases with TOH more than 14 days (TOH was 15, 15, and 16 days, respectively), the lymphocyte subset alterations in convalescence of COVID-19 patients were not seen in this study. More studies including patients with TOH more than 14 days need to investigate to observe lymphocyte subset alterations in whole natural history of the disease.

## Conclusions

Present study revealed independent predictors for severe COVID-19 and found CD4^+^ T cell was mainstay of immunity response to severe SARS-CoV-2 infection. T lymphocyte and its subset negatively correlated with disease severity, CT manifestation and delayed hospitalization. The counts of lymphocyte subset were changed more profound than their proportions. These findings would provide some new insights in management of COVID-19.

## Data Availability

The datasets generated and/or analyzed during the current study are available from the corresponding author on reasonable request.
